# Aid alignment for global health research: the role of HIROs

**DOI:** 10.1186/1478-4505-9-12

**Published:** 2011-03-17

**Authors:** Roderik F Viergever

**Affiliations:** 1 Department of Global Health and Development, London School of Hygiene and Tropical Medicine, London, UK

## Abstract

The lack of a mechanism that aligns financial flows for global health research towards public health priorities limits the impact of health research on health and health equity. Collaborative groups of health research funders appear to be particularly well situated to ameliorate this situation and to initiate discussion on aid alignment for global health research. One such group is the Heads of International Research Organizations (HIROs), which brings together a large number of major government and philanthropic funders of biomedical research. Surprisingly, there is hardly any information publicly available on HIROs' objectives, or on how it aims to achieve more harmonization in the field of research for health. Greater transparency on HIROs' objectives and on its current efforts towards addressing the gap between global health research needs and investments would be desirable, given the enormous potential benefits of more coordination by this group.

## Introduction

"There is no global coordination of research and development for major diseases, and the global health research and innovation system is highly fragmented" [1]. Such was one of the conclusions of the report of the World Health Organization (WHO) Expert Working Group on Research and Development Financing that was finalized in the past year. This conclusion is not novel; it has been consistently argued for decades that the lack of a harmonized approach for prioritizing, funding, and planning research for health has prevented that research from responding adequately to the world's health needs [2-4]. To this day, the allocation of health research funding exhibits little tendency to be commensurate with burden of disease [5], and only a small percentage of funding is allocated towards research that addresses the health problems of developing countries, an issue often referred to as the 10/90 gap [2,6]. The harmful consequences of this funding gap for everyday clinical practice in developing countries are extensive. For instance, in 2006 it was shown that 30 years of pharmaceutical research had resulted in the development of only 21 drugs targeting neglected diseases (including malaria and tuberculosis) out of a total 1556 new chemical entities marketed [7]. Although the emergence of public-private partnerships, increases in total expenditure on global health research and in the number of actors engaged in that research, and a shift in epidemiology of disease in low- and middle-income countries have all substantially changed the landscape of health research for development in recent years, the enduring mismatch between health research needs and investments remains a cause for grave concern [6,8].

## Discussion

The Global Ministerial Forum on Research for Health in Bamako in 2008, in line with the Paris Declaration and the Accra Agenda for Action [9], recognized the need for increased coordination in the field of research for health and the important role of research funding institutions therein [10]. It called on funders of research and innovation "to better align, coordinate, and harmonize the global health research architecture and its governance". There are several collaborative groups of funders of research for health that appear to be well situated to achieve this. One such group is the Heads of International Research Organizations (HIROs). HIROs was established more than ten years ago and brings together government and philanthropic funding institutions for biomedical research, including major funders such as the US National Institutes of Health and the Bill and Melinda Gates Foundation [1]. The enormous collective influence of these organizations is apparent, but is also demonstrated by the impact external donors have been shown to have on WHO's budget allocations [11] and by research showing that a small group of eleven organizations (many of which are a part of HIROs) currently provide 75% of global funding for neglected disease research and development [5,12]. Besides HIROs, there are other groups where funders of research for health collaborate. Especially the 'Enhancing Support for Strengthening the Effectiveness of National Capacity Efforts' initiative (ESSENCE) - a collaborative framework between funding agencies to scale up research capacity and increase the effectiveness of research for health in Africa - has recently made some encouraging first steps in aligning donor funding towards national priorities for health research [13]. Other examples are the Product Development Partnership (PDP) Funders group (formerly known as the PDP Donor Coordination Group), whose purpose is to facilitate donors in supporting and monitoring the performance of PDPs [14]; the International Forum of Research Donors (IFORD), which brings together funders of research related to international development [15]; and the International Health Partnership+ (IHP+), which aims to improve the impact of health aid in general [16].

In discussing the need for increased coordination among funders of research for health, it is important to consider what exactly needs to be coordinated. Recent positive developments among funders include the identification of common approaches to monitoring and evaluation and sharing research data [17,18]. However, funders have been found to fall short of agreeing on a harmonized agenda for research funding [19]. This finding is worrying; in order to maximize the impact of research investments on health and health equity it is of fundamental importance that funders agree on common health research priorities, both in countries and on the global level, and act on those priorities in a coordinated manner [20-23].

Since HIROs brings together the heads of major funders of biomedical research, it appears to be particularly well suited to give rise to the major changes in health research governance that are called for by the Bamako call to action. Unfortunately, HIROs has made little information available on its goals or on how it aims to achieve increased harmonization, alignment and coordination. An internet search reveals only websites noting that a meeting has taken place, and a search on PubMed for *"Heads of International Research Organisations" OR "Heads of International Research Organizations" OR "Heads of International biomedical Research Organisations" OR "Heads of International biomedical Research Organizations" OR HIRO[Title/Abstract] OR HIROs[Title/Abstract] *returns no relevant results. HIROs is not the only group where funders collaborate that is sparing with information. Recently, IHP+ was criticized for its lack of transparency [24]. Individual funders have also been criticized for not being transparent enough in their operations [25].

## Conclusion

An initiative like the HIROs group is most welcome in the crowded field of global health research funders. It is surely one of the few groups that could initiate discussion on aid alignment for global health research. Given the enormous potential benefits of more coordination by this group, the contents of its discussions are of great interest to the global health research community. More transparency on HIROs' intentions for achieving increased coordination and on its current efforts towards addressing the gap between global health research needs and investments would therefore be desirable.

## Competing interests

The authors declare that they have no competing interests.
